# The effect of Neuragen PN^® ^on Neuropathic pain: A randomized, double blind, placebo controlled clinical trial

**DOI:** 10.1186/1472-6882-10-22

**Published:** 2010-05-20

**Authors:** Li Li

**Affiliations:** 1 Department of Kinesiology, Louisiana State University, Baton Rouge, LA 70803, USA

## Abstract

**Background:**

A double blind, randomized, placebo controlled study to evaluate the safety and efficacy of the naturally derived topical oil, "Neuragen PN^®^" for the treatment of neuropathic pain.

**Methods:**

Sixty participants with plantar cutaneous (foot sole) pain due to all cause peripheral neuropathy were recruited from the community. Each subject was randomly assigned to receive one of two treatments (Neuragen PN^® ^or placebo) per week in a crossover design. The primary outcome measure was acute spontaneous pain level as reported on a visual analog scale.

**Results:**

There was an overall pain reduction for both treatments from pre to post application. As compared to the placebo, Neuragen PN^® ^led to significantly (p < .05) greater pain reduction. Fifty six of sixty subjects (93.3%) receiving Neuragen PN^® ^reported pain reduction within 30 minutes. This reduction within 30 minutes occurred in only twenty one of sixty (35.0%) subjects receiving the placebo. In a break out analysis of the diabetic only subgroup, 94% of subjects in the Neuragen PN^® ^group achieved pain reduction within 30 minutes vs 11.0% of the placebo group. No adverse events were observed.

**Conclusions:**

This randomized, placebo controlled, clinical trial with crossover design revealed that the naturally derived oil, Neuragen PN^®^, provided significant relief from neuropathic pain in an all cause neuropathy group. Participants with diabetes within this group experienced similar pain relief.

**Trial registration:**

**ISRCTN registered: **ISRCTN13226601

## Background

The incidence of peripheral neuropathy is growing alongside the epidemic of type 1 and type 2 diabetes [1]. Use of prescription medication to manage this condition is often limited to analgesics that can be associated with long term dependency and side effects, making these less desirable treatments from a patient's standpoint [2].

Current pharmaceutical treatments of neuropathic pain include anticonvulsants, antidepressants and opioids. For example, the American Society of Pain Educators has released Consensus Treatment Guidelines for the management of diabetic peripheral neuropathy (DPN) pain. A board of 11 pain specialists has published the recommendations in a three-tier fashion in the Proceedings of the Mayo Clinic [3]. The first tier group of drugs to treat DPN includes duloxetine, oxycodone, pregabalin, and the tricyclic antidepressants. Duloxetine and Pregabalin are oral prescription medications approved by the FDA to treat peripheral neuropathic pain [4]. Duloxetine has a myriad of adverse side effects such as dose dependent blood pressure increases and multiple drug interactions [5]. Relatively common side effects of pregabalin include dizziness, dry mouth, and coordination problems. The second tier drugs include the anticonvulsants carbamazepine and gabapentin. These drugs are also associated with a variety of side effects including marked sedation, dizziness, and dry mouth. It is therefore important to consider novel approaches to treating neuropathic pain that may have less potential side effects.

Neuragen PN^® ^is an FDA registered homeopathic drug containing a blend of six homeopathic substances and five plant based essential oils. The six homeopathic substances are St. John's Wort (*Hypericum perforatum*), Wolfsbane (*Aconitum napellus*), Club Moss (*Lycopodium clavatum*), phosphorus, Poison Ivy (*Rhus toxicodendron*) and Rye ergot (*Secale cornutum*). Their potencies are all 12C, and all six of these substances have a record of traditional use for nerve related pain (Homeopathic reperatories list them as moderately or strongly recommended for: Generalities, Injuries, nerves; Generalities, Pain, burning or shooting) [6]. Although homeopathic substances are often delivered as single agents (i.e. one substance per dose), there are a number of clinical studies that have employed a combination of homeopathic substances within one product (i.e. multiple substances per dose) for various disorders [7-10]. Research has also shown that terpenes and sequiterpenes (found in plant based essential oils) can enhance skin permeation. The five essential oils in Neuragen PN^®^, a proprietary blend of geranium oil (*Pelargonium graveolens*), lavender oil (*Lavandula angustifolia*), bergamot oil (*Citrus aurantium*), tea tree oil (*Melaleuca alternifolia*) and eucalyptus oil (*Eucalyptus globulus*), act as transdermal carriers. These carriers are believed to assist in the penetration of the homeopathic ingredients through the stratum corneum of the skin [11,12].

This study was conducted in the Department of Kinesiology at Louisiana State University (LSU) over a two week period during the spring of 2008 to examine the analgesic effects and safety of Neuragen PN^®^.

## Methods

### Participants

Individuals with physician diagnosed peripheral neuropathy and pain levels greater than 3 but no higher than 8 (on a 0-10 visual analog scale, VAS, of present pain intensity) were recruited through existing University databases, community support groups, and advertisements placed in community newsletters and newspapers. People with a pain level greater than 8 were excluded based on our pilot study which revealed that subjects had difficulty distinguishing between pain levels of 9 and 10. Pain at level 10 is labelled as "Worst possible pain", which often is a state insensitive to actual pain reduction. Potential participants were screened according to predetermined inclusion and exclusion criteria (Table 1). Prior to participation, eligible participants read and signed an informed consent form. The project was approved by the Lousiana State University Institutional Review Board.

**Table 1:** Inclusion and Exclusion Criteria

Inclusion Criteria	Exclusion Criteria
At least 21 years of age.	Pregnancy.

Established diagnosis of neuropathic pain for at least the past 3 months, confirmed by primary care physician and/or neurologist.	Previous use, continuing use, or other knowledge of Neuragen PN^®^.

Pain of at least level 3 and no higher than 8 on a 0-10 scale, despite other treatments.	Evidence of other types of pain as, or more severe, than the pain under study.

Normal cognitive and communication skills (as judged by investigator) and ability to complete self report questionnaires.	Major psychological conditions requiring treatment.

	History of eczema/atopy/anaphylaxis or unusual skin reactions.

	Self reported sensitivity to perfumes, essential oils, or strong odors.

	Changes to current pain management regime within the previous month.

### Pain Assessment

Self reported pain was assessed by the Short Form McGill Pain Questionnaire [13]. Present Pain Intensity (PPI) was reported on a 10 cm Visual Analog Scale (VAS) ranging from "No Pain" to "Severe Pain." Participant instructions were to "Mark the line at the point related to the severity of pain you are currently experiencing in your feet." VAS scores were calculated by determining the distance between the left hand side of the scale (i.e., No Pain) and the mark. Distances were rounded to the nearest centimeter to produce a score from 0-10. Numerical rating scales have been widely used in pain research and have been demonstrated to be capable of identifying clinically meaningful changes [14].

### Protocol

Participants completed two experimental sessions separated by one week. Previous experience indicated that the clinical effects of Neuragen^® ^typically last approximately 8 hrs and not more than 24 hrs. A one week "washout" was therefore determined to be an adequate period of time to minimize carryover effects. Upon arriving at the LSU lab, participants received 10 minutes of seated rest before completing the Short Form McGill Pain Questionnaire (Pre-Treatment). One of two treatments (described below) was then given according to a balanced design, which was performed by the blinded investigator. This design ensured that all participants would receive both treatments by the end of the investigation. Half of the subjects would receive the active treatment first and the other half would receive the placebo treatment first. Following 30 minutes of additional seated rest (post-treatment), PPI was reassessed using the McGill questionnaire.

Participants then returned to their normal daily activities, but were first outfitted with a personal digital assistant (PDA) preprogrammed with the Purdue Momentary Assessment Tool (PMAT, Bangstate, Inc.). This software enabled digital presentation of the McGill questionnaire at specific time points throughout the day. At each time point, an audio reminder prompted the participant to answer the questions using the standard PDA touch screen format. At each time point, the participant was given a 5 minute window to begin answering the questionnaire. If the participants did not start answering the questions within this window, it was disabled until the next time point. This procedure ensured that participant responses were given at specific times from initial pain assessment (2, 3, 4, 5, 6, 7, 8 and 9 hours).

Participant responses were stored on the PDA and uploaded to a computer during each participant's subsequent visit. For each time point the VAS score was calculated. It is of note that due to screen size limitations, the digitally presented VAS was shorter than that of the VAS on paper. The software divided its VAS (~ 4 cm) into ten equal units, and participant responses were determined using similar methods to those of the paper version.

At each testing session, participants were given an adverse event report form and instructed to fill it out to the best of their ability if they experienced any abnormal reaction to the treatment.

### Treatment

Treatments in the study consisted of two topical oils: "Neuragen PN^®^", a topical oil containing homeopathic and plant extract ingredients, and a placebo consisting of USP light mineral oil with 5% v/v cis rose oxide added to approximate the odor of the active treatment. Researchers and participants were blinded to treatment, as each was labeled only as "Neuragen A" or "Neuragen B". The treatments were labeled by the manufacturer, who in turn did not reveal the nature of the Neuragen A or B designations to the investigators until after the data had been analyzed. Treatments were applied by the researcher to the skin of the participant's feet. The oil was administered from a spray bottle with each spray covering the area with a thin film. One spray was applied to the top of the foot aimed at the base of the toes, another spray to the top of the foot half way between the toes and the ankle, one spray to the internal side of the foot aimed at the middle, one spray to the external side of the foot aimed at the middle, and one spray to the underside of the foot aimed at the ball of the foot. The total amount sprayed was approximately 0.75 ml per foot per treatment.

Neuragen PN^® ^has a unique floral odor due to its volatile botanical constituents. It was not possible to replicate the odor of Neuragen PN^® ^exactly in the placebo without replicating its analgesic effects. However, since participants were treated only in a clinical setting, the investigator masked the odors of the treatments by distributing (e.g. spraying) Neuragen PN^® ^in the treatment room before application. With the floral odor permeating the room, investigators and subjects could not distinguish which treatment they were giving or receiving. This methodology also controlled for any potential mechanism of action involving the effects of fragrance inhalation without topical application to the skin, since all participants were exposed to an approximately equivalent level of odor. Also, any participants with previous knowledge of Neuragen PN^® ^were excluded from the study, since they may have been aware of an association between odor and efficacy.

### Statistical Analysis

Test-retest reliability of pre-test Short Form McGill Pain Questionnaire scores (SPRI, APRI, Total PRI, VAS, and Overall Intensity of Total Pain Experience) was assessed by Intraclass Correlation Coefficients (ICC) [15]:(1)

Where  and ; DF - degree of freedom for error term related to the independent variables of "subject", different "test", "repeat" and the overall "error" term.

Two separate two factor analyses of variance (ANOVA) with repeated measures were completed to examine the short term (Pre-treatment and Post-treatment) and long term (2 - 9 hour) effects of the two treatments. Tukey's post hoc analysis was employed whenever necessary (*P *< .05 observed in main effects or interactions). Significance level was set at alpha = .05.

## Results

### Participants

**Figure 1:**
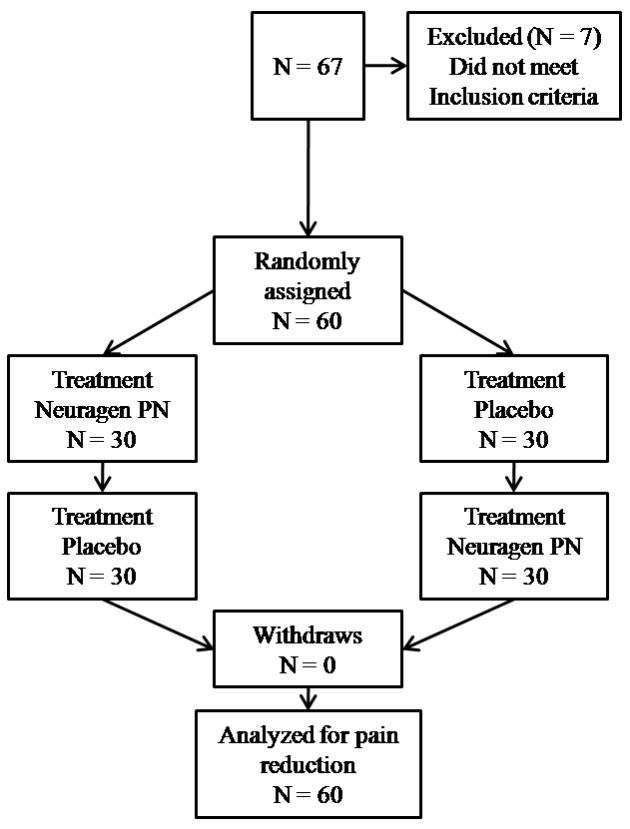
**Trial outline**.

Sixty-seven individuals with physician diagnosed peripheral neuropathy were recruited from the community and screened for eligibility. Of these individuals, sixty met all predetermined inclusion criteria and were included in the study. All participants completed the intervention in two different testing sequences in order to balance the potential order effects (See Figure 1 for details). Participant demographics, peripheral neuropathy duration and etiology, and use of concurrent oral analgesics has been presented in Table 2.

**Table 2:** Trial participants that met the inclusion criteria

Men	n = 24
Women	n = 36

Age (years)	69 ± 10

Height (cm)	167 ± 9

Body Mass (kg)	83 ± 21

PN duration (years)	7 ± 5

Diabetes induced PN	n = 18

Chemotherapy induced PN	n = 2

Idiopathic induced PN	n = 32

Other causes of PN (ie. Trauma)	n = 8

0 Concurrent Analgesics	21

1 Concurrent Analgesic	30

2 Concurrent Analgesics	6

3 Concurrent Analgesics	3

>3 Concurrent Analgesics	0

### Effects of Neuragen PN^®^

All of the items in the McGill Pain Questionnaire responded similarly to the experimental treatments but the VAS provided the most quantitative assessment. The VAS was therefore chosen as the representative criterion measure due to its high test-retest reliability and widespread use in clinical research.

**Figure 2:**
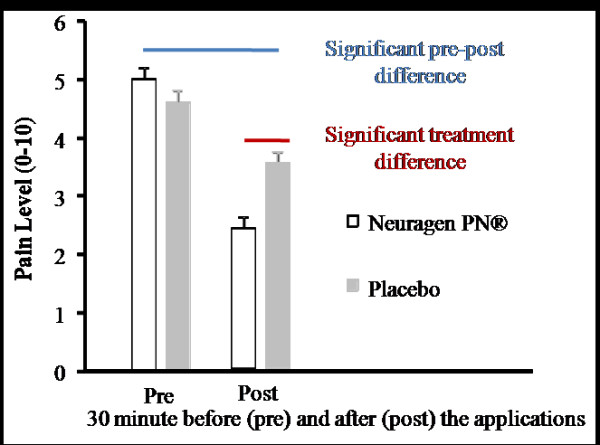
**Average pain reduction effects of Neuragen PN^® ^and the placebo, represented by the Visual Analog Scale (VAS), are graphed with a time (pre/post) axis**. The VAS ranged from 0-10. The vertical whiskers on each data point represent the standard error about that point. Pre/post pain level was measured 30 minutes before/after treatment applications. There was no significant difference in pain levels before the treatment applications. There was an overall pain reduction for both treatments from pre to post application. Further, Neuragen PN^® ^had significantly greater pain reduction effects than the placebo (p < .05)

There was no significant order effect observed due to different testing sequences. The results were pooled and only treatment effects are reported here. Pain reduction effects of Neuragen PN^® ^and the placebo were measured using the VAS 30 minutes before (Pre) and after (Post) treatment application. The results are shown in Figure 2. There was no significant difference in pain levels before the treatment applications. There was an overall pain reduction, indicated by the VAS, for both treatments from pre to post application. Further, Neuragen PN^® ^had significantly greater pain reduction effects than the placebo (*P *< .05).

Table 3 summarizes the pain relieving effects of each treatment based only on Pre and Post test scores. Fifty-six of sixty subjects (93.3%) receiving Neuragen PN^® ^reported pain reduction within 30 minutes. This reduction within 30 minutes occurred in only twenty-one of sixty (35.0%) subjects receiving the placebo. The number of subjects exhibiting a 30% reduction in pain or greater was 83% of patients receiving Neuragen PN^® ^vs 22% receiving the placebo. In addition, 52% of patients receiving Neuragen PN^® ^achieved a reduction in pain scores of at least 50% compared with 3% of patients receiving the placebo.

**Table 3:** VAS pain results for Neuragen PN^® ^and the placebo where SE stands for standard error of the mean.

Treatment	Time	Mean	SE
Neuragen PN^®^	Pre	4.7	0.2

Placebo	Pre	4.2	0.2

Neuragen PN^®^	Post	2.53	0.2

Placebo	Post	3.98	0.2

There was an overall pain reduction for both treatments from pre to post application. Further, Neuragen PN^® ^had significantly greater pain reduction effects than the placebo (p < .05)

**Figure 3:**
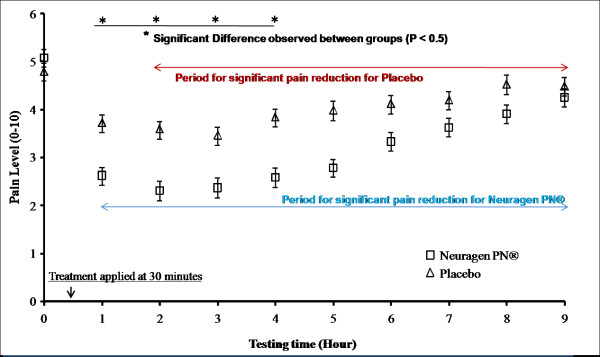
**Average pain reduction effects of Neuragen PN^® ^and the placebo, represented by the Visual Analog Scale (VAS) where 0 indicates no pain and 10 indicates maximal or severe pain**. The vertical whiskers on each data point represent the standard error about that point. Pre pain level was measured 30 minutes (hour 0) before treatment application and post pain was measured 30 minutes (hour 1) after treatment application and every hour thereafter (hour 2 - 9), up to 8 hours. There was a pain reduction (p < .05) for both treatments up to hour 9. Neuragen PN^® ^led to more pain reduction than the pacebo during the first 4 hours after treatment application

Figure 3 shows the pain reduction effects of the two different treatments over a 9 hour period.

Of the 18 participants with diabetes, 94% reported a reduction in pain within 30 minutes of the application of Neuragen PN^® ^compared with only 11% of the participants receiving the placebo. In a sub group analysis, 78% of the participants with diabetes receiving Neuragen PN^® ^experienced a reduction of pain scores of at least 30% versus only 11% of participants with diabetes treated with the placebo. In addition, a reduction of pain scores of at least 50% was experienced by 56% of participants with diabetes receiving Neuragen PN^® ^and only 6% of participants with diabetes receiving the placebo.

## Discussion

A randomized, double blind placebo controlled clinical trial was conducted to assess the efficacy of the Neuragen PN^®^, a topical analgesic formula, in the treatment of neuropathic pain. This topical oil was tested for its ability to relieve self-reported foot pain as measured by a visual analog pain scale. Neuragen PN^® ^*and *the placebo resulted in reduced self-reported pain in as little as 30 minutes. Neuragen PN^® ^treatment resulted in pain reduction over and above that caused by the placebo (p < .05), however treatment pain reduction was not significantly different from the placebo between the hours of 5 and 9.

Peripheral neuropathy related pain levels were tracked for approximately eight hours following application. Importantly, the use of portable PDAs equipped with the Purdue Momentary Assessment Tool (PMAT, Bangstate, Inc.) allowed participants to leave the laboratory and return to their normal activities of daily living. This instrumentation also ensured that *all *participant responses were given on an hourly basis following treatment application (within five minutes). Reported pain levels (2 - 9 hours) therefore reflect those that may occur under normal, everyday situations. One limitation of the results is the small number subjects (n = 60). Another possible limitation is that the presentation of the VAS on the PDA was smaller than its presentation on the paper format. The difference on the absolute scale may influence the subjects' selection of their subjective pain level. Therefore, comparison of the data collected on paper (ie. immediate effects VAS (pre and post)) with data collected from the PDAs (2 - 9 hours) should be done with caution. In addition, no effort was made to assess pain relief beyond the reporting period mentioned, or to assess the long term effects of repeated dosing. Future trials are planned to address these issues.

More than 50% of people who suffer from diabetes have (or will develop) diabetic peripheral neuropathy [16]. Treatment of diabetic peripheral neuropathy usually consists of a prescription for gabapentanoids, antidepressants, or opioid analgesics. These prescription medications have all been found to have a limited success rate and they are also associated with long term use side effects [2,17]. Although the mechanism of action of Neuragen PN^® ^is not known at this time, its efficacy and good safety profile recommend it's use as another option in the pain management of peripheral neuropathies.

## Conclusion

Other than capsaicin [18,19], no clinical trials were found in the literature that investigate the pain relieving effect of naturally derived over the counter topical treatments for neuropathic pain. The literature does contain studies of a number of naturally derived compounds, such as Cannabinoids [20] and Conotoxins from predatory snails [21] which are currently in prescription drug development for the treatment of neuropathic pain. The results of this Neuragen PN^® ^clinical trial therefore contribute to the study of pain reduction by expanding the spectrum of this type of inquiry. We also believe we are the first to use PDAs to record pain levels on an hourly basis. This method is easy to use, reliable and accurate. The use of new technology also contributes to the methodology development of pain management related studies.

This clinical trial revealed that the naturally derived oil, Neuragen PN^®^, provided significant relief from neuropathic pain. Pain relief was statistically significant for up to 8 hours, and the use of Neuragen PN^® ^resulted in 52% of subjects (vs 3% of the placebo group) receiving a maximal pain relief of 50% or greater (*P *< .05) within 30 minutes of application. Additionally, 56% of the diabetic subgroup receiving Neuragen PN^® ^(vs. 6% of participants with diabetes in the placebo group) experienced a maximal pain relief of 50% or greater (*P *< .05) within 30 minutes of application. As there were no adverse events reported during the study period, Neuragen PN^® ^is recommended as a safe and efficacious treatment to provide temporary relief from neuropathic pain.

**ISRCTN registered: **ISRCTN13226601

## Competing interests

The study is funded by Origin Biomed Inc. http://originbiomed.com/. Origin Biomed has participated in study design, decision to publish, and preparation of the manuscript. However, Origin Biomed did not take part in the data collection, data analysis, or interpretation and discussion of the results.

## Pre-publication history

The pre-publication history for this paper can be accessed here:

http://www.biomedcentral.com/1472-6882/10/22/prepub
